# Travel bubble policies for low‐risk air transport recovery during pandemics

**DOI:** 10.1111/risa.14348

**Published:** 2024-06-24

**Authors:** Yaoming Zhou, Siping Li, Tanmoy Kundu, Tsan‐Ming Choi, Jiuh‐Biing Sheu

**Affiliations:** ^1^ Department of Industrial Engineering & Management Shanghai Jiao Tong University Shanghai China; ^2^ Operations Management & Quantitative Techniques Area Indian Institute of Management Indore Indore India; ^3^ Centre for Supply Chain Research, Management School University of Liverpool Liverpool UK; ^4^ Department of Business Administration National Taiwan University Taipei Taiwan

**Keywords:** air transport recovery, network design, pandemic, risk analysis, travel bubble policies

## Abstract

Global pandemics restrict long‐haul mobility and international trade. To restore air traffic, a policy named “travel bubble” was implemented during the recent COVID‐19 pandemic, which seeks to re‐establish air connections among specific countries by permitting unrestricted passenger travel without mandatory quarantine upon arrival. However, travel bubbles are prone to bursting for safety reasons, and how to develop an effective restoration plan through travel bubbles is under‐explored. Thus, it is vital to learn from COVID‐19 and develop a formal framework for implementing travel bubble therapy for future public health emergencies. This article conducts an analytical investigation of the air travel bubble problem from a network design standpoint. First, a link‐based network design problem is established with the goal of minimizing the total infection risk during air travel. Then, based on the relationship between origin‐destination pairs and international candidate links, the model is reformulated into a path‐based one. A Lagrangian relaxation‐based solution framework is proposed to determine the optimal restored international air routes and assign the traffic flow. Finally, computational experiments on both hypothetical data and real‐world cases are conducted to examine the algorithm's performance. The results demonstrate the effectiveness and efficiency of the proposed model and algorithm. In addition, compared to a benchmark strategy, it is found that the infection risk under the proposed travel bubble strategy can be reduced by up to 45.2%. More importantly, this work provides practical insights into developing pandemic‐induced air transport recovery schemes for both policymakers and aviation operations regulators.

## 1 INTRODUCTION

Air transportation plays a vital role in facilitating global connectivity (Gong et al., 2023; Wang et al., 2020), while it also faces nontrivial risk, especially under pandemics such as COVID‐19 (Xu et al., 2023). The heightened infection risk is compounded by the rapid global spread of the virus through international air travel, thus amplifying the challenges in implementing effective containment and mitigation strategies (Brockmann & Helbing, 2013; Nicolaides et al., 2020). Consequently, many travel restrictions, such as flight suspension, on‐arrival quarantine, and national lockdown, are implemented to control the disease spread, which poses difficulties for the development of global aviation and tourism industries (Kajitani et al., 2023; Zhou et al., 2021). Particularly in the recent COVID‐19 pandemic, the world has witnessed the unprecedented phenomenon that “no one can travel from one country to another.” In this context, airlines worldwide suffered tremendous losses as their fleets remain grounded, and countless hotels and tourist destinations stood empty for a very long time (Sun et al., 2021). Although the COVID‐19 pandemic was basically in “the past,” the severe economic impact caused by COVID‐19 induces us to rethink the delicate balance between public health and traffic recovery. Therefore, it is indispensable for governments and policymakers to investigate how to develop international cooperation and innovative solutions in cross‐border transportation for potential future pandemics.

To open pathways for air transport recovery, a new concept called “travel bubble” was generated during the COVID‐19 pandemic. Travel bubbles are considered to be a closed‐loop system, meaning passengers can fly freely within countries in the bubble. The travel website Wego provides a comprehensive definition of the travel bubble: “*Travel bubbles are essentially an exclusive partnership between two or more countries to restore connections by opening up borders and allowing people to travel freely without having a quarantine period*.”^1^ Table 1 lists some well‐documented travel bubble practices during the COVID‐19 pandemic.

**TABLE 1:** Travel bubble practices and their implemented time.

Countries (regions)	Implemented time
India with the United States, France, and Germany^a^	July 2020
Japan with Cambodia, Laos, Malaysia, Myanmar, and Taiwan^b^	September 2020
Vanuatu and New Caledonia^c^	February 2021
Australia and New Zealand^d^	April 2021
The UAE with Seychelles, Greece, Serbia, and Bahrain^e^	May 2021
Singapore and Hong Kong^f^	May 2021
South Korea with Saipan and Singapore^g^	October 2021
Indonesia and Singapore^h^	January 2022

^a^ Explained: What are “air bubbles” in international travel, who can use and benefit from India's new agreements. https://www.indiatoday.in/india/story/explained‐what‐are‐air‐bubbles‐in‐international‐travel‐who‐can‐use‐1701918‐2020‐07‐18 (accessed March 26, 2023).

^b^ Japan has confirmed its travel bubble with five Asian regions—here are the entry rules. https://www.timeout.com/tokyo/news/japan‐has‐confirmed‐its‐travel‐bubble‐with‐five‐asian‐regions‐here‐are‐the‐entry‐rules‐091420 (accessed March 26, 2023).

^c^ Vanuatu and New Caledonia launch travel bubble. https://www.stuff.co.nz/travel/destinations/pacific‐islands/300236047/vanuatu‐and‐new‐caledonia‐launch‐travel‐bubble (accessed March 26, 2023).

^d^ Quarantine free flights resume between Australia and New Zealand. https://airport‐world.com/flights‐resume‐between‐australia‐and‐new‐zealand/ (accessed March 26, 2023).

^e^ UAE expands list of “safe travel” corridors as it eyes post‐Covid tourism recovery. https://www.arabianbusiness.com/industries/travel‐hospitality/463319‐uae‐expands‐list‐of‐safe‐travel‐corridors‐as‐it‐eyes‐post‐covid‐tourism‐recovery (accessed March 26, 2023).

^f^ Singapore and Hong Kong to open travel bubble. https://www.bbc.com/news/business‐56883766 (accessed March 26, 2023).

^g^ Travel bubble triples Koreans’ trips to Saipan. https://www.kedglobal.com/travel‐leisure/newsView/ked202110100003 (accessed March 26, 2023).

^h^ Indonesia‐Singapore travel bubble: What you need to know. https://jakartaglobe.id/news/indonesiasingapore‐travel‐bubble‐what‐you‐need‐to‐know (accessed March 26, 2023).

It can be seen that the concept of “travel bubble” presents several advantages for moving toward a “new normal” during pandemics. On the one hand, by allowing people to move across borders, it offers a certain convenience for various activities such as family reunions, leisure travel, and business negotiations. Taking New Zealand as an example, after establishing the Trans‐Tasman travel bubble, the total border crossings were well up compared with the earlier months of 2021.^2^ On the other hand, the implementation of travel bubbles has the potential to expedite economic recovery. By facilitating the flow of travelers in a controlled and safe manner, destinations within the bubble can experience a bounce in tourism‐related revenue. According to a report at the beginning of the Trans‐Tasman travel bubble, it was estimated that up to NZ$1 billion could be brought to the New Zealand economy (for the rest of that calendar year) by the opening of the travel bubble.^3^ The International Air Transport Association states that the travel bubble policy offers a flexible solution with public health risk mitigation measures. It can be developed to restart international aviation.^4^ Note that there are some other policies implemented to promote air traffic recovery during the COVID‐19 pandemic, such as vaccination passports, slot liberalization, and government subsidies. However, these policies are from the perspective of the stakeholders involved in air travel, and they do not essentially reduce the in‐transit infection risk through comprehensive restoration schemes. Additionally, a prerequisite for these policies is to first establish a safe and low‐risk environment, which is the goal of implementing travel bubbles. In short, the travel bubble policy has been a viable method for air traffic recovery during pandemics, and it stands out for its direct focus on mitigating the in‐transit infection risk.

However, many travel bubbles taking off in the COVID‐19 pandemic finally burst with regret, mainly because the traveling environment could not remain low‐risk. Sun et al. (2023) indicate that establishing a travel bubble is like “walking a tightrope.” An arbitrarily designed travel bubble would expose passengers to high infection risks, so the pandemic situation in the bubble would become not optimistic. On the contrary, overly restrictive schemes would impact mobility and trade between partners, and thus, the international travel demand cannot be fully satisfied with limited resources. To overcome these difficulties, it is essential to establish a formal framework for devising a low‐risk air transport recovery scheme and easing cross‐border restrictions through air travel bubbles. Drawing from the definition of risk in risk science as “*the consequences of an activity and associated uncertainties*” (Aven, 2024; Logan et al., 2021), we can express risk as (*A,C,U*), where events (*A*) can result in consequences (*C*) with uncertainties (*U*) (Johnson et al., 2021; Logan et al., 2022). In the context of our study, risk pertains to the activity of air transport recovery during pandemics. Event *A* represents the implementation of travel bubble policies, potentially resulting in consequence *C*, wherein passengers face for being infected during air travel. Uncertainty *U* denotes that before operating an air travel bubble scheme, we do not know whether the traveling environment will be safe for passengers. Therefore, motivated by the advantages of travel bubbles and the risk management challenges in their implementation during pandemics, we seek to address the following three research questions:

(1) What factors influence the implementation of the bilateral air travel bubble scheme? How do they affect the optimal choices for implementing an air travel bubble scheme between countries?
(2) Can the air travel bubble help fulfill the possible travel demands during a pandemic situation? If yes, how can aviation practitioners and policymakers reconfigure (or design) the air transport network (ATN) with limited resources (bilateral air routes)?
(3) What is the optimal recovery scheme for air traffic using travel bubbles? Does the above intervention help control the infection risk while ensuring a safe air travel recovery?

This article proposes a risk‐oriented optimization modeling framework for the travel bubble policy from a network design perspective. The aim is to help policymakers and aviation operations practitioners plan and evaluate decisions on restoring international air routes and designing the ATN during pandemics. Our study supposes that the leading country establishes air bubbles with several partner countries for bilateral travel. Hence, there are two subnetworks: one is the current domestic ATN of the leading country, and the other is the ATN comprised of the existing air routes and operating airports of its partner countries. We can find that the core of the air travel bubble problem (ATBP) is to reconnect the two subnetworks to satisfy all specific travel demands during the pandemic. Therefore, we formulate the ATBP as a transportation network design problem that minimizes the total infection risk during air travel subject to many operational constraints. In addition, the solution algorithms based on Lagrangian relaxation are developed for efficiently seeking out the planning for international air traffic restoration. To the best of our knowledge, this article positions itself as the first study in the literature on transportation to formulate and address the ATBP using optimization models for determining the recovery scheme during pandemics. It is worth mentioning that although the travel bubble policy was proposed and implemented during the recent COVID‐19 pandemic, it is a potential and feasible solution for low‐risk air transport recovery in future global catastrophes.

The contributions of this article are three‐fold. First, the ATBP is formulated as a novel risk‐oriented network design model that considers the link connection of separate networks with minimal infection risk. Moreover, the mathematical model is further reformulated into an analytically simpler one based on the relationship between origin‐destination (OD) pairs and international candidate links. Second, a Lagrangian relaxation‐based solution framework is proposed to solve the ATBP model‐based optimization problem, which decomposes the ATBP into a set of independent small‐size problems that can be conveniently handled. The presented algorithm can efficiently solve large‐scale problems with an explicit small optimality gap. Third, this article provides policymakers with a powerful decision‐support tool to implement the air travel bubble strategy during pandemics. Hence, we believe that the insights not only contribute to the literature but also advance the real‐world practices related to the design and implementation of the air travel bubble.

The rest of this article is arranged as follows. Section 2 reviews the relevant literature on our topic, and Section 3 presents a detailed description of the ATBP. After that, a link‐based network design model based on the classical traffic assignment problem is proposed to characterize the ATBP in Section 4, and it is reformulated as a path‐based model according to the problem feature. Section 5 develops a solution framework based on Lagrangian relaxation, including solving the relaxed problem, finding the feasible solution, and updating Lagrange multipliers. Then, Section 6 provides an airport risk evaluation approach based on the entropy weight method (EWM). A series of computational experiments are conducted to demonstrate the proposed method's effectiveness and efficiency in Section 7. Theoretical and practical implications are discussed in Section 8, followed by conclusions and future research in Section 9.

## 2 LITERATURE REVIEW

To clearly show this study's literature positioning and research gaps, we review the relevant literature on travel bubble policy and ATN design.

### 2.1 Travel bubble policy

The recent COVID‐19 pandemic has given rise to many new research topics on healthcare, social, economic, and political problems (Choi, 2021; Gupta et al., 2022; Zhang et al., 2023). In addition, it seriously influences numerous transportation systems (Choi & Shi, 2024; Jiang, 2021; Li et al., 2023) and supply chains (Li & Zhou, 2024; Niu et al., 2023; Xu et al., 2023). The “travel bubble” is one of the most representative new concepts that emerged during this pandemic. Due to its nascent nature, few systematic studies exist. In general, current studies in this area can be divided into two categories: (1) identification and evaluation of this policy and (2) discussion of its rationality.

The first set of studies focuses on how travel bubbles exist and what the generated impacts are. One of the representative studies in this part is Sun et al. (2022), which successfully addressed the identification of travel bubbles in actual flight data. They defined a flight ratio discrepancy to investigate up to which degree the concept of travel bubbles exists for air transportation. Linka et al. (2020) applied the SEIR model to study the safety of travel bubbles during the COVID‐19 pandemic. It was shown that partial reopening within local travel bubbles is a reasonable first step for recovery. Li et al. (2021) proposed a network‐based method to examine the international passenger air transport connectivity of a particular country in the presence of travel bubbles. The critical countries affecting international connectivity are also identified for prioritizing the candidate regions to form travel bubbles. Chen et al. (2023) employed an automatic emotion analysis method to examine people's attitudes to travel bubble policies in social media. They analyzed some real data and found that cooperative travel arrangements improve the confidence of residents in air travel.

The other group of studies discusses the rationality of implementing travel bubble policies. Sharun et al. (2020) analyzed the implications and risks associated with travel bubbles, emphasizing that travel bubbles are beneficial to facilitating trade and economic recovery. Still, the establishment and efficient maintenance will be a challenge for decision‐makers. Luo and Lam (2020) investigated the relationships among the fear of infection, the anxiety of traveling, risk preference, and the intention of traveling to a travel bubble destination. The authors found that the level of infection fear would not reduce people's travel intention. Yu et al. (2021) discussed people's willingness to participate in the travel bubble policy through a survey. They found that the travel bubble strategy is successful because participants look forward to traveling and understand how to prevent infection. Shen et al. (2022) commented that the bubble strategy is a feasible solution to let international travel restore to normal in the complex COVID pandemic world.

Indeed, drawing lessons and deriving insights from the COVID‐19 pandemic are imperative for both governments and airlines, especially concerning the establishment and management of travel bubbles (Fusté‐Forné & Michael, 2023; Shaw & Scully, 2023). The emphasis on passenger safety and perception during air travel has proven to be a key tool in achieving air traffic restoration (Cxallı & Cxallı, 2023; Girish et al., 2023). In this context, some intelligent analytical methods have emerged to improve the overall passenger experience (Srinivas & Ramachandiran, 2024). Additionally, by identifying passenger perception and coordinating response efforts during emergencies, stakeholders can enhance the resilience of aviation operations management and mitigate the negative impact of potential future pandemics (Li & Wang, 2024; Siegrist et al., 2021).

### 2.2 ATN design

Air transport involves risk (Chung et al., 2017; Sun et al., 2020), so the rational ATN design is very pivotal. The design of ATNs is a branch of the “network design problem” (NDP), which aims to find an optimal allocation and utilization of resources to obtain a specific goal in transportation planning. Magnanti and Wong (1984) presented a general version of the NDP, and they gave some solution algorithms for the proposed model. Yang and Bell (1998) comprehensively reviewed the models and algorithms of the NDP, especially for road network design. They classified the NDP into three types, namely, the “discrete NDP,” the “continuous NDP,” and the “hybrid NDP.” The NDP has been applied in many areas, including facility location (Cui et al., 2010; Iliopoulou & Kepaptsoglou, 2019), logistics management (Liu et al., 2022; Wang et al., 2019), and disaster response (Gomez et al., 2019; Zhang et al., 2023).

The application of the NDP to air transportation was first explored by O'Kelly (1986), in which airports in the ATN were divided into hub nodes and spoke nodes, where hub nodes were fully interconnected, while spoke nodes could only connect to hub nodes. Several models under different assumptions were developed, and numerical results were reported using a 25‐city data set in the United States. Skorin‐Kapov et al. (1996) gave a detailed description of uncapacitated single and multiple hub location problems. The proposed model was solved by heuristic algorithms and tested on the same data set. Lederer and Nambimadom (1998) analyzed the network choice for airlines by using a stylized model, including expenses for airlines and passengers. They found that “hub‐and‐spoke networks” would have high “schedule frequency” while low “schedule reliability,” while the direct service is the opposite. Büdenbender et al. (2000) introduced a direct flight network design problem where several airports must be connected by direct transportation. They developed a “hybrid tabu search and branch‐and‐bound algorithm” to solve practical instances within acceptable computation times. Lin et al. (2012) presented a capacitated hub location model with economies of scale and integral constraints on the paths. They used a genetic algorithm to solve the model and implemented numerical experiments on the Chinese air cargo network. Intending to build reliable air‐travel infrastructures, Deshpande and Arikan (2012) analyzed the impact of network structure on flight delays. The results showed that the “hub‐and‐spoke network structure” significantly impacts the “scheduled on‐time arrival probability.” An et al. (2015) studied the reliable hub‐and‐spoke design problem. In their model, each potential hub airport has a fixed disruption probability, and the selection of backup hubs and alternative routes is considered. Shen et al. (2021) proposed a reliable air transportation network design model under random disruptions. They developed a method to compute the distributional information regarding network disruptions and applied tractable mixed‐integer linear programming to portray the problem. Zhao et al. (2023) explored the distributionally robust chance‐constrained models for the p‐hub center problem. The authors proposed a constraint‐generation approach to speed up the solution process. They conducted computational experiments to test the proposed network design. Furthermore, Sindhwani et al. (2024) developed a viable hub location model, considering both social and economic objectives. They numerically demonstrated that their method can improve the regional connectivity of ATNs and mitigate the impact of disruption risks.

In addition to the above literature oriented toward the strategic design of ATNs, some previous studies focus on the schedule planning and dynamic adjustment of ATNs at the operational stage. Lan et al. (2006) developed two new approaches to optimize aircraft routings and flight departure times to minimize passenger disruptions. Using data from a major U.S. airline, the computational results showed that the presented robust plan can significantly reduce the number of passenger misconnections. Erdelyi and Topaloglu (2010) proposed a theoretical model to find the optimal decisions on capacity allocation and overbooking over an ATN. The authors utilized a state aggregation approach to obtain high‐quality solutions and conducted several computational experiments to show the advantage of the proposed model. Wei et al. (2014) introduced the flight routes addition/deletion problem to optimize the algebraic connectivity of ATNs. They presented three methods, the modified greedy perturbation, the weighted tabu search, and the relaxed semidefinite programming to solve the problem and discussed their applicable conditions. Wang and Jacquillat (2020) explored stochastic ATN operations with the goal of optimizing scheduling interventions and ground‐holding operations across ATNs. An efficient algorithm using new dual integer cuts and incorporating original neighborhood constraints is designed to solve the largest test instances. Chen et al. (2022) focused on examining the optimization problem for “air taxi operations” in a city region. They presented a grid‐based hub location model and designed a novel variable neighborhood search heuristic to solve this problem. A comprehensive review of the “air taxi operations” for urban mobility can be found in the work of Rajendran and Srinivas (2020). Ding et al. (2023) applied a novel deep reinforcement learning method to the flight network‐based airline disruption recovery problem. They showed that the proposed framework can obtain efficient and cost‐effective recovery solutions. Finally, Manchiraju et al. (2023) analyzed the impact of network changes and operational improvements on the on‐time performance of airlines. They found that the dynamic adjustment at the operational stage is significant, while the changes to network structure are constrained by many other factors.

### 2.3 Research gaps

In summary, several research gaps regarding the implementation of travel bubble policies can be drawn from the existing literature.

First, most previous studies on travel bubble policies concentrate on the effects of their implementation, and no mathematical models and solution methods for the restoration scheme are available in the literature. Since travel bubble policies have a favorable impact on economic development during pandemics, designing a low‐risk travel bubble is a big concern for both policymakers and aviation operations regulators.

Second, the ATN is typically viewed as a whole when conducting network design, and the connections between subnetworks are hardly considered. However, country‐to‐country disconnections in the ATN are very common during pandemics. The reconnection of international air routes is necessary for moving toward a “new normal.” Thus, it is crucial to explore the connections between subnetworks for air traffic restoration.

Third, prior studies in the extant literature on ATN design focus on the location of hub airports and the disruptions at airports. However, airports are not disrupted during pandemics, while air routes between countries are frequently interrupted and resumed. Moreover, the in‐transit risk has not been considered in network design. Therefore, the existing methodology is not sufficient to meet the requirements of travel bubble policies.

To fill the above‐mentioned research gaps, we propose in this article a risk‐oriented network design model to depict this ATBP. Our model can simultaneously determine the restored international air routes between disconnected subnetworks and assign the passenger flow for all OD pairs. A solution technique based on Lagrangian relaxation is developed to solve the model efficiently. Furthermore, we apply the proposed models and algorithms to real‐world ATNs. The findings can help policymakers and aviation operations regulators when implementing the travel bubble policy for low‐risk air transport recovery.

## 3 PROBLEM DESCRIPTION

**FIGURE 1:**
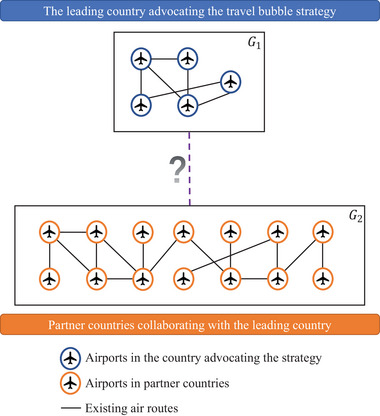
Illustration of the ATBP.

The ATBP involves a leading country advocating the policy and some partner countries follow and collaborate with the leading country. As illustrated in Figure 1, the existing ATN consists of two subnetworks $G_{1}=G\left(V_{1},E_{1}\right)$ and $G_{2}=G\left(V_{2},E_{2}\right)$, where *G*_1_ denotes the connections between airports in the leading country, while *G*_2_ represents the connections between airports in its partner countries. The international air routes between this country and its partner countries are known as the candidate links connecting *G*_1_ and *G*_2_, which are operated at the prepandemic stage but suspended due to the outbreak of the pandemic. Therefore, the problem consists of simultaneously selecting this country's restored international air routes and determining the assignment of traffic flow to satisfy the specific traffic demand between each OD pair during the pandemic period.

Considering the unique nature of pandemics like COVID‐19, the most important factor influencing the restoration scheme is the infection risk. Hence, our proposed model aims to minimize the total infection risk that passengers may encounter. In the ATBP, each existing and potential air route carries a risk value quantified by several pandemic‐related decision‐making indices, and the method for determining this risk value is presented in Section 6. The other two key factors are budgetary resources and traffic demand, which are coupled with each other. The budgetary resources represent the number of restored air routes, while the traffic demand denotes the potential demand for international air travel. Under air travel bubble policies, the suspended air routes are considered to be partly recovered to meet the current international traffic demand. Therefore, the core of the ATBP is to determine which potential routes can be restored to satisfy the demands with the lowest infection risk.

## 4 MATHEMATICAL MODEL

### 4.1 A link‐based network design model

From the problem description, a link‐based network design model (M1) is proposed based on the classical traffic assignment problem (Beckmann et al., 1956; Gao et al., 2005; Peeta & Ziliaskopoulos, 2001; Wardrop, 1952).

Definition 1 (Link). A link is defined as a direct connection between two nodes of the network.

Definition 2 (Path). A path is defined as a sequence of distinct nodes from origin to destination, where a direct link connects each pair of nodes.

Let $E=E_{1}\cup E_{2}\cup E_{3}$ be the set of links, where *E*_1_ and *E*_2_ are the set of existing links in *G*_1_ and *G*_2_, respectively, and *E*_3_ is the set of international candidate links between *G*_1_ and *G*_2_. *I* consists of all international OD pairs at the prepandemic stage and *K_i_* is the set of all possible paths connecting an OD pair *i*. Key notations used in this model are presented in Table 2.

**TABLE 2:** Key notations used in the link‐based network design model.

Category	Notation	Meaning
Sets	*E*	Set of links
	*E*_1_	Set of existing links in *G*_1_
	*E*_2_	Set of existing links in *G*_2_
	*E*_3_	Set of international candidate links between *G*_1_ and *G*_2_
	*I*	Set of OD pairs
	*K_i_*	Set of paths connecting OD pair *i*
Indices	*a*	Index of links, *a* ∈ *E*
	*i*	Index of OD pairs, *i* ∈ *I*
	*k*	Index of paths connecting OD pair *i*, *k* ∈ *K_i_*
Parameters	**d_a_**	The vector of pandemic‐related decision‐making indices of link *a*
	*r_a_* (**d_a_**)	The risk value for one unit flow on link *a*
	*q_i_*	The total traffic demand of OD pair *i* during the pandemic period
	$\delta_{a,k}^{i}$	$\delta_{a,k}^{i}=1$ if link *a* is on path *k* of OD pair *i*, and 0 otherwise.
	*c_a_*	The capacity of link *a* during the pandemic period
	*T*	The maximum number of links on each path
	*B*	The number of restored links
Decision variables	*u_a_*	*u_a_* = 1 if link *a* is restored, and 0 otherwise.
	*x_a_*	The total flow on link *a*
	$m_{k}^{i}$	$m_{k}^{i}=1$ if path *k* of OD pair *i* is used, and 0 otherwise.
	$f_{k}^{i}$	The traffic flow on path *k* of OD pair *i*

With these notations, the link‐based model (M1) can be formulated for the ATBP with the aim of minimizing the total infection risk *R* exposed to the passengers on all existing and candidate links in the ATN:

(1) $$\min\;R=\sum_{a\in E}r_{a}\left(d_{a}\right)x_{a},$$

where the risk value for one unit flow on each link is determined by a function of its pandemic‐related decision‐making indices. It is quantified by the relative possibility of being infected for the traveler on a specific link, using the EWM, as will be discussed in Section 6. Viewed through the lens of risk science, the total risk represents the expected consequences, namely, the infection of travelers caused by the event of implementing the air travel bubble.

Next, six groups of constraints are developed to satisfy the requirements mentioned in Section 3.

(1) Traffic assignment constraints

According to the classical traffic assignment problem, the traffic demand of each OD pair should be served by its possible paths. Thus, the constraints are formulated as:

(2) $$\sum_{k\in K_{i}}f_{k}^{i}=q_{i}\;\forall i\in I.$$

(2) Flow conservation constraints

The second set of constraints ensures the flow conservation between links and paths. In other words, the traffic flow on one link is equal to the sum of traffic flow on paths passing through this link for all OD pairs:

(3) $$x_{a}=\sum_{i\in I}\sum_{k\in K_{i}}f_{k}^{i}\delta_{a,k}^{i}\;\forall a\in E.$$

(3) Constraints between binary variables and flow variables of candidate links

It is necessary to prohibit the flow on any candidate link that is not restored because only the restored links can be used in the ATN. Therefore, the following constraints must hold:

(4) $$x_{a}\leq c_{a}u_{a}\;\forall a\in E_{3}.$$

Remark 1 Note that constraints (4) also state that the carrying flow of the candidate links cannot exceed their capacity limit. However, the capacity constraints do not appear on the links in $G_{1}$ and $G_{2}$. The reason is that the traffic flow brought by the travel bubble policy only occupies a small proportion of their capacity.

(4) Constraints between binary variables and flow variables of paths

Similar to constraints (4), we introduce a large constant M (i.e., the “big M”) to connect the relationship between binary variables $m_{k}^{i}$ and flow variables $f_{k}^{i}$:

(5) $$f_{k}^{i}\leq M*m_{k}^{i}\;\forall i\in I,k\in K_{i}.$$

(5) Passenger transfer constraints

In air travel, passengers do not want to make too many stopovers to reach their destination. Hence, this set of constraints represents that passengers at most can take T flights from the origin to the destination:

(6) $$m_{k}^{i}*\sum_{a\in E}\delta_{a,k}^{i}\leq T\;\forall i\in I,k\in K_{i}.$$

(6) Budget constraint

Considering that the amount of recovery resources is limited in the ATBP, the following budget constraint stipulates the number of restored links:

(7) $$\sum_{a\in E_{3}}u_{a}=B.$$

From the formulations mentioned above, the link‐based model (M1) can be summarized as follows:

$$\min\;R=\sum_{a\in E}r_{a}\left(d_{a}\right)x_{a},$$

s.t. Constraints (2)‐(7).

(8) $$u_{a}=\left\{0,1\right\}\;\forall a\in E_{3}.$$

(9) $$m_{k}^{i}=\left\{0,1\right\}\;\forall i\in I,k\in K_{i}.$$

(10) $$f_{k}^{i}\geq 0\;\forall i\in I,k\in K_{i}.$$

It can be seen that M1 is a mixed integer linear programming (MILP) problem with four types of decision variables. However, solving M1 is difficult due to the large number of variables and constraints in this model. This is an important issue as we explain below. As opposed to road networks, where nodes are only connected to a few neighboring nodes (Zhou et al., 2022), airports in ATNs are usually connected to many other airports. Thus, there are multitudes of possible paths between a given OD pair in ATNs. For instance, the number of possible paths between ATL (Hartsfield Jackson Atlanta International Airport) and JFK (John Fitzgerald Kennedy International Airport) in the ATN of the United States is over 10,000,000 (Barabási, 2016). We can find that the numbers of binary variables $m_{k}^{i}$ and flow variables $f_{k}^{i}$ in M1 are very large. To the best of our knowledge, the model with variables on the scale of tens of million is challenging to solve using commercial solvers. Therefore, it is necessary to convert M1 into a simplified model according to the features of the ATBP.

### 4.2 Model reformulation: A path‐based model

In this section, we transform M1 into a path‐based model based on the relationship between path and link. It can be found that there is one and only one international candidate link on each path of a given OD pair. Thus, the following proposition is presented.

Proposition 1 *Given an OD pair and an international candidate link that can connect this OD pair, the path with the lowest risk is unique, and the traffic flow should be assigned to this path*.

For any given OD pair i∈I and a candidate link $l\in E_{3}$ which can connect this OD in the ATN, we let (1) $K_{i}^{l}=\left\{p_{1},p_{2},\text{…},p_{|K_{i}^{l}|}\right\}\subseteq K_{i}$ be the set of possible paths of the OD pair i including link l, and (2) 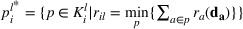 be the path with the lowest risk in $K_{i}^{l}$. For each unit flow from the origin to the destination via link l, it must choose the path with the lowest risk. This leads to the traffic flow on other paths 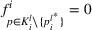. Therefore, we have found an optimal plan for all paths simply by assigning the traffic flow to the path with the lowest risk.□

Proposition 1 implies that, given an OD pair, there are at most $|E_{3}|$ choices of the flow assignment from the origin to the destination, where $|E_{3}|$ is the number of international candidate links. Hence, we denote the fraction of the traffic demand from OD pair i∈I that uses the international candidate link $l\in E_{3}$ by $z_{il}$. The corresponding unit risk is denoted by $r_{il}$, while the number of links on this path is denoted by $t_{il}$. We let 
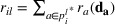
 and 
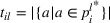
 based on Proposition 1, where 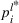 is the path with the lowest risk. With these preparations, M1 can be converted into a new path‐based model M2. Key notations used in M2 are presented in Table 3.

**TABLE 3:** Key notations used in the path‐based network design model.

Category	Notation	Meaning
Sets	$E_{3}$	Set of international candidate links between $G_{1}$ and $G_{2}$
	I	Set of OD pairs
Indices	l	Index of international candidate links, $l\in E_{3}$
	i	Index of OD pairs, i∈I
Parameters	$r_{il}$	The risk value for one unit flow on the path where OD pair i uses the international candidate link l
	$q_{i}$	The total traffic demand of OD pair i during the pandemic period
	$c_{l}$	The capacity of link l during the pandemic period
	$t_{il}$	The number of links on the path where OD pair i uses the international candidate link l
	T	The maximum number of links on each path
	B	The number of restored links
Decision variables	$u_{l}$	$u_{l}=1$ if link l is restored, and 0 otherwise.
	$m_{il}$	$m_{il}=1$ if the path where OD pair i uses the international candidate link l is employed, and 0 otherwise.
	$z_{il}$	The fraction of the traffic demand from OD pair i that uses the international candidate link l

Therefore, the path‐based model (M2) can be formulated as follows:

(11) $$\min\;R=\sum_{i\in I}\sum_{l\in E_{3}}q_{i}r_{il}z_{il},$$

(12) $$s.t.\;\sum_{l\in E_{3}}z_{il}=1\;\forall i\in I.$$

(13) $$\sum_{i\in I}q_{i}z_{il}\leq c_{l}\;\forall l\in E_{3}.$$

(14) $$m_{il}\leq u_{l}\;\forall i\in I,l\in E_{3}.$$

(15) $$z_{il}\leq m_{il}\;\forall i\in I,\;l\in E_{3}.$$

(16) $$t_{il}m_{il}\leq T\;\forall i\in I,\;l\in E_{3}.$$

(17) $$\sum_{l\in E_{3}}u_{l}=B.$$

(18) $$u_{l}=\left\{0,1\right\}\;\forall l\in E_{3}.$$

(19) $$m_{il}=\left\{0,1\right\}\;\forall i\in I,l\in E_{3}.$$

(20) $$0\leq z_{il}\leq 1\;\forall i\in I,l\in E_{3}.$$

In M2, the objective function (11) minimizes the total risk, which is the sum of the risk of each possible path. Constraints (12) are the demand constraints that correspond to constraints (2) in M1. Constraints (13) limit the carrying flow of each candidate link, and constraints (14) and (15) contact three types of decision variables $u_{l}$, $m_{il}$, and $z_{il}$. They jointly state the meaning of constraints (4) and (5) in M1. The passenger transfer constraints and the budget constraint are shown in (16) and (17), whose implications are consistent with constraints (6) and (7) in M1. Constraints (18)‐(20) indicate the domains of decision variables.

Proposition 2 *M1 is equivalent to M2*.

By inserting constraints (3) into the objective function (1), we obtain the following:

(21) $$\begin{array}{ccc} R & = & \sum_{a\in E}r_{a}\left(d_{a}\right)\sum_{i\in I}\sum_{k\in K_{i}}f_{k}^{i}\delta_{a,k}^{i}\\ & = & \sum_{i\in I}\sum_{k\in K_{i}}\sum_{a\in E}f_{k}^{i}r_{a}\left(d_{a}\right)\delta_{a,k}^{i}. \end{array}$$

Since $\delta_{a,k}^{i}$ denotes whether link a is on path k of OD pair i, we can remove the term where $\delta_{a,k}^{i}=0$ and rewrite the objective function in a path‐based format:

(22) $$R=\sum_{i\in I}\sum_{k\in K_{i}}f_{k}^{i}\sum_{a\in k}r_{a}\left(d_{a}\right).$$

Note that there is one and only one international candidate link on each path of a given OD pair. Thus, paths can be distinguished by different candidate links:

(23) $$R=\sum_{i\in I}\sum_{l\in E_{3}}\sum_{p\in K_{i}^{l}}f_{p}^{i}\sum_{a\in p}r_{a}\left(d_{a}\right).$$

From Proposition 1, we know that for given an OD pair i∈I and a candidate link $l\in E_{3}$, the traffic flow should be assigned to the path 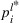 with the lowest risk, and 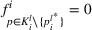. Thus, the objective function can be converted into:

(24)

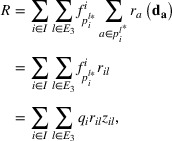

which is the objective function of M2. We can see that M2 utilizes the flow conservation restriction between paths and links in M1 to transform the objective function, and the other constraints have the same meaning as those in M1. Therefore, M1 can be equivalently stated as M2.□

Compared with M1, the number of variables and constraints in M2 is significantly reduced. Thus, in developing algorithms to solve the ATBP, we seek to utilize the path‐based model M2 that minimizes the total infection risk based on each possible path. It should be noted that M1 is inspired by (but different from) the classical traffic assignment problem. In particular, the objective function aims to minimize the total infection risk on all links. Based on the relationship between the international candidate link and the path of OD pairs, M1 can be transformed into M2, which minimizes the infection risk of all selected paths.

Proposition 3 *The ATBP is NP‐hard*.

See Appendix A.□

Proposition 3 suggests that there is little likelihood of using a deterministic algorithm that runs in polynomial time to solve the ATBP. Consequently, it is necessary to design efficient algorithms for solving the ATBP in a reasonable amount of time. We discuss this important issue in Section 5.

## 5 SOLUTION ALGORITHMS BASED ON LAGRANGIAN RELAXATION

In this section, we employ Lagrangian relaxation to develop the solution algorithms for the ATBP. The Lagrangian relaxation technique often yields good solutions within a reasonable computational time (Fisher, 2004; Pirkul & Schilling, 1998), and it has been successfully applied in many studies on network design problems (An et al., 2015; Bell et al., 2020; Tong et al., 2015). In the Lagrangian relaxation procedure, we need to: (1) relax some constraints and incorporate them into the objective function through multiplying by Lagrange multipliers, (2) solve this relaxed problem under the fixed values of Lagrange multipliers, and (3) find a feasible solution of the primal problem from the relaxed solution and update multipliers. The detailed algorithm design is as follows.

### 5.1 Solving the relaxed problem

One critical problem of applying Lagrangian relaxation is to decide which constraints in the model should be relaxed. For the ATBP, we find that constraints (12) dominate “hard” constraints because they strictly stipulate the fulfillment of the traffic demand of each OD pair. Thus, we relax the demand constraints (12) with the corresponding Lagrange multipliers $\lambda_{i}$. Although the relaxation of constraints (12) indicates that the demand of each OD pair does not have to be tightly satisfied, we need to ensure that the total capacity from the selected international links is sufficient to serve the demand of all OD pairs. In this way, the restored links selected in the relaxed problem can be used to find a feasible solution to the primal problem. Therefore, we add a total capacity constraint (25) to the relaxed problem:

(25) $$\sum_{i\in I}q_{i}\leq \sum_{l\in E_{3}}c_{l}u_{l}.$$

Then, we obtain the model M3 of the relaxed problem:

(26) $$\max_{\lambda }\min_{U,Z}\;\sum_{i\in I}\sum_{l\in E_{3}}\left(q_{i}r_{il}-\lambda_{i}\right)z_{il}+\sum_{i\in I}\lambda_{i},$$

s.t. Constraints (13)‐(20), and (25).

Under the fixed values of $\lambda_{i}$, it can be seen that the second term of the objective function is a constant. Thus, we focus on solving the relaxed problem to minimize the first term of the objective function.

To solve this problem, let us recall the relationship between the candidate link and the potential path. Based on constraints (14) and (15), if a candidate link $l\in E_{3}$ is not restored ($u_{l}=0$), no traffic flow will be assigned to paths where this link is located ($m_{il}=0$ and $z_{il}=0$ for all OD pairs i∈I). It means this link contributes 0 to the objective function (26). On the contrary, if this link is selected for restoration, its contribution to the objective function (26) is determined by the following subproblem:

(27) $$\min\;S_{l}=\sum_{i\in I}\left(q_{i}r_{il}-\lambda_{i}\right)z_{il},$$

(28) $$s.t.\;\sum_{i\in I}q_{i}z_{il}\leq c_{l}.$$

(29) $$z_{il}\leq m_{il}\;\forall i\in I.$$

(30) $$t_{il}m_{il}\leq T\;\forall i\in I.$$

(31) $$m_{il}=\left\{0,1\right\}\;\forall i\in I.$$

(32) $$0\leq z_{il}\leq 1\;\forall i\in I.$$

Remark 2 It is worth mentioning that the coefficient $\left(q_{i}r_{il}-\lambda_{i}\right)$ and the traffic demand $q_{i}$ of each OD pair i∈I are crucial to solve this subproblem. If the coefficients $\left(q_{i}r_{il}-\lambda_{i}\right)$ in the objective function (27) are negative, the task is to select OD pairs i∈I, which have large negative coefficients along with small traffic demand for consuming the capacity in constraints (28). On the contrary, if the coefficients are positive, we will not select these OD pairs.

Hence, we define a contribution ratio $n_{il}$ for each OD pair i∈I, which is essential for solving this subproblem.

Definition 3 (Contribution ratio). The contribution ratio is the value that the OD pair i∈I contributes to the objective function per unit consumption of the link capacity. It is written as the coefficient $\left(q_{i}r_{il}-\lambda_{i}\right)$ in the objective function (27) divided by its traffic demand $q_{i}$:

(33) $$n_{il}=\frac{q_{i}r_{il}-\lambda_{i}}{q_{i}}.$$

With this contribution ratio, the subproblem for each candidate link $l\in E_{3}$ can be solved by Algorithm 1.

ALGORITHM 1 **Algorithm for solving the subproblem (27)‐(32).**

**Table:** 

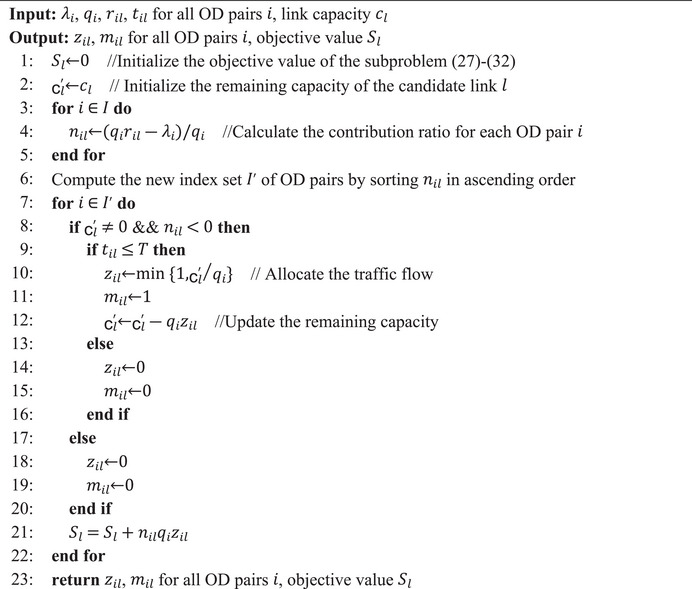

Based on Algorithm 1, the value of $S_{l}$ for all candidate links can be obtained. Then, we can determine the value of variables $u_{l}$ by solving the following integer programming problem:

(34) $$\min\;\sum_{l\in E_{3}}S_{l}u_{l},$$

(35) $$s.t.\;\sum_{i\in I}q_{i}\leq \sum_{l\in E_{3}}c_{l}u_{l}.$$

(36) $$\sum_{l\in E_{3}}u_{l}=B.$$

(37) $$u_{l}=\left\{0,1\right\}\;\;\;l\in E_{3}.$$

In this problem, the objective function (34) has the same meaning as the first term of the objective function (26). Constraints (35) and (36) are the total capacity constraint and the budget constraint, respectively, which are the same as constraints (25) and (17). Constraints (37) define the domain of binary variables $u_{l}$. It is clear that if the total capacity of the candidate links with the B smallest values of $S_{l}$ exceeds the total traffic demand, the problem can be solved by setting the corresponding $u_{l}$ to 1. Otherwise, we need to solve this problem using the branch‐and‐bound technique. In detail, the solution process of the Lagrangian relaxed problem is presented in Algorithm 2.

ALGORITHM 2 **Algorithm for solving the Lagrangian relaxed problem.**

**Table:** 

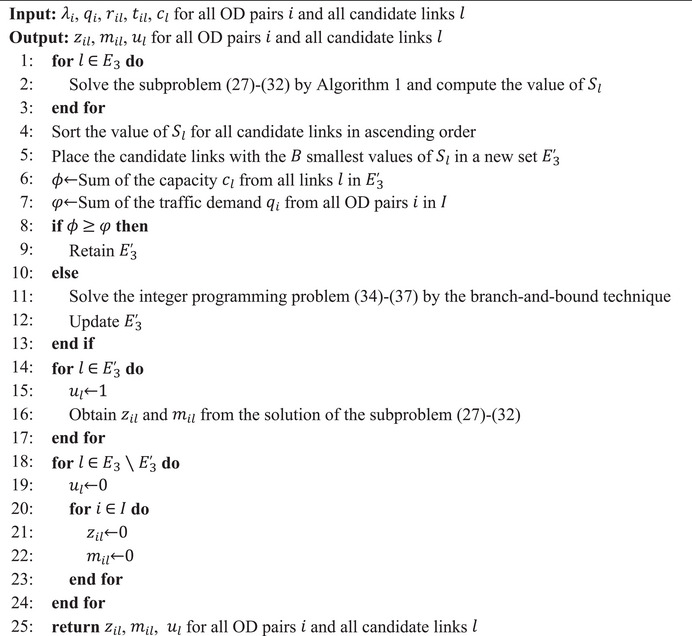

In each iteration, the decision variables $u_{l}$, $m_{il}$, and $z_{il}$ of the relaxed problem can be found according to Algorithm 2, and the objective value serves as a lower bound on the optimal value of the primal problem. It can be seen that the solution obtained by Algorithm 2 might not be globally optimal because some heuristic rules are taken in deciding the restored links. However, it is not necessarily required that the solution of the relaxed problem in each iteration is optimal. The reason is that this lower bound is used to obtain the solution of the primal problem during the iteration process. Moreover, Algorithm 2 can at least get a near‐optimal solution quickly, thus reducing the time spent in solving the relaxed problem. We denote this lower bound by $LB^{n}$, where n is the current iteration index. In addition, we let LB be the largest lower bound that we have found so far. It is worth mentioning that the solution here is not likely to be feasible for the primal problem due to the relaxation of constraints (12). Therefore, we need to find a feasible solution to the primal problem depending on the relaxed solution.

### 5.2 Finding a feasible solution and updating multipliers

In order to obtain a primal feasible solution, we present Proposition 4, which can exploit the solution of the relaxed problem.

Proposition 4 *The ATBP can be converted into an unbalanced transportation problem when the restored international links are fixed*.

We let $E_{3}^{\prime }\subset E_{3}$ be the set of restored international links satisfying $|E_{3}^{\prime }|=B$. Then, the binary variables $u_{l}$ are known. If $u_{l}$ are feasible to the total capacity constraint, the optimal assignment of traffic flow to the restored international links can be obtained by solving the following model M4:

(38) $$\min\;\sum_{i\in I}\sum_{l\in E_{3}^{\prime }}q_{i}r_{il}z_{il},$$

(39) $$s.t.\;\sum_{l\in E_{3}^{\prime }}z_{il}=1\;\forall i\in I.$$

(40) $$\sum_{i\in I}q_{i}z_{il}\leq c_{l}\;\forall l\in E_{3}^{\prime }.$$

(41) $$z_{il}\leq m_{il}\;\forall i\in I,l\in E_{3}^{\prime }.$$

(42) $$t_{il}m_{il}\leq T\;\forall i\in I,l\in E_{3}^{\prime }.$$

(43) $$m_{il}=\left\{0,1\right\}\;\forall i\in I,l\in E_{3}^{\prime }.$$

(44) $$0\leq z_{il}\leq 1\;\forall i\in I,l\in E_{3}^{\prime }.$$

If we consider I as the set of supply centers (sources) and $E_{3}^{\prime }$ as the set of receiving centers (destinations) in M4, this problem can be regarded as an unbalanced transportation problem with an excess demand capacity, where the decision variables $z_{il}$ represent the fraction of the commodity distributed from source i∈I to destination $l\in E_{3}^{\prime }$, and the decision variables $m_{il}$ restrict the distances from supply centers to receiving centers from being too long. In this case, let the variables $m_{il}$ and $z_{il}\left(\forall i\in I,l\in E_{3}\setminus E_{3}^{\prime }\right)$ in M2 be 0 since the corresponding international candidate links are not selected.□

With this property, if we are given the fixed values of $u_{l}$, the decision variables $m_{il}$ and $z_{il}$ can be determined by solving a transportation problem. The transportation problem is a well‐known problem with mature solution methods in operations research, and it can be solved efficiently in software. Thus, we fix the international links selected in the relaxed problem and then solve the corresponding transportation problem (38)‐(44) to get the traffic flow assignment. The objective value of this transportation problem provides an upper bound for the primal problem. Similarly, we denote this value computed on the *n*th iteration by $UB^{n}$, and mark the smallest upper bound over all current iterations in the procedure by UB.

The Lagrange multipliers are iteratively updated using the standard subgradient optimization procedure (Fisher, 2004; Sherali & Myers, 1988). We begin by computing a step‐size $t^{n}$ on the *n*th iteration as follows:

(45) $$t^{n}=\Delta \left[\frac{UB-LB^{n}}{\sum_{i\in I}{\left(1-\sum_{l\in E_{3}}z_{il}^{n}\right)}^{2}}\right],$$

where Δ is a constant named as the step‐size multiplier, and $z_{il}^{n}$ is the value of the corresponding variable $z_{il}$ on the *n*th iteration. Then, the Lagrange multipliers on the (n+1) th iteration are revised according to the following equation:

(46) $$\lambda_{i}^{n+1}=\max\left\{0,\lambda_{i}^{n}+t^{n}\left(1-\sum_{l\in E_{3}}z_{il}^{n}\right)\right\}.$$

It should be noted that the step‐size multiplier Δ will be replaced by Δ/2, and the Lagrange multipliers will be reset to the values that obtain the current largest lower bound LB if there is no improvement in LB within some consecutive iterations. The specific values of such parameters for computational experiments are described in Section 7.

**FIGURE 2:**
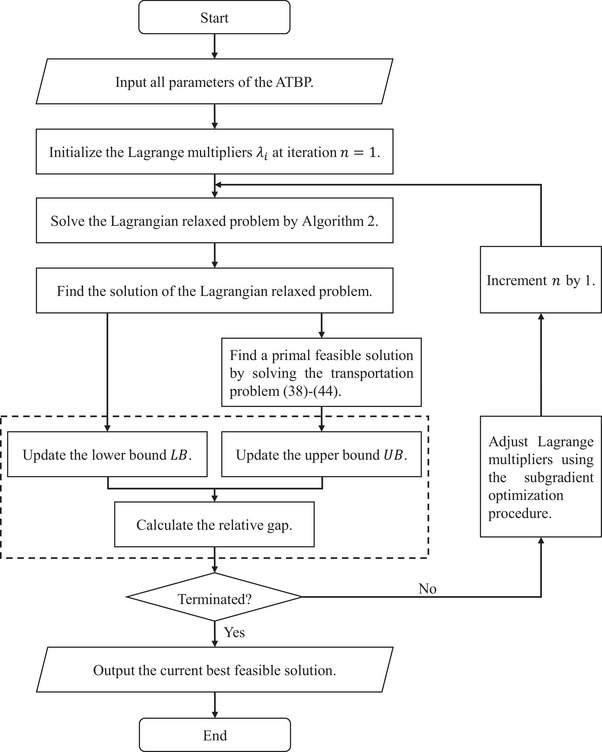
Flow chart of the Lagrangian relaxation‐based solution algorithm for solving the ATBP.

The Lagrangian relaxation procedure is terminated when one of the following two conditions is fulfilled. One is that the lower bound LB is sufficiently close to the upper bound UB. We set an optimality tolerance ε and stipulate the relative gap $\frac{UB-LB}{UB}\leq \varepsilon$. The other is that the number of iterations reaches the prespecified maximum number. Putting together the above parts, the overall algorithm framework to solve the ATBP is presented in Figure 2.

## 6 AIRPORT RISK EVALUATION BASED ON THE ENTROPY WEIGHT METHOD

As discussed in the introduction, risk comprises three fundamental components, event (A), consequence (C), and uncertainty (U). Given the event of implementing travel bubble policies, it is required to handle the uncertainty for representing the airport risk, which reflects the potential local epidemic situation passengers may encounter and serves as an essential input for the expected consequence. To quantitatively evaluate the risk, we employ the EWM. The EWM, grounded in Shannon entropy, is a well‐established objective evaluation technique extensively utilized in risk assessment (Liu et al., 2017; Mishra & Ayyub, 2019). It quantifies uncertainty in information, and its formulation is deeply rooted in probability theory (Huang et al., 2021). Thus, the EWM can facilitate the measurement for uncertainty. In our evaluation, we first select some pandemic‐related decision‐making indices influencing air travel safety as risk indicators and then determine the weight of each indicator based on the EWM.

Definition 4 (Airport risk). The airport risk is defined as its comprehensive score in the EWM, which is the sum of the product of the indicator weight and the normalized indicator value of this airport.

Definition 5 (Air route risk). The air route risk is defined as the higher risk of two ending airports.

To quantify the local epidemic situation, three risk indicators^5^ are chosen to evaluate the airport risk, namely, the infection rate, case fatality rate, and vaccination rate in cities where airports are located. After collecting the data of all risk indicators, the specific steps of the EWM are summarized as follows:

(1) Formulate the initial data as an information decision matrix D:
(47) $$D=\left[\begin{array}{ccc} d_{11} & d_{12} & d_{13}\\ d_{21} & d_{22} & d_{23}\\ \vdots & \vdots & \vdots \\ d_{i1} & d_{i2} & d_{i3}\\ \vdots & \vdots & \vdots \\ d_{m1} & d_{m2} & d_{m3} \end{array}\right],$$

where m is the total number of airports in the air travel bubble strategy.

(2) Normalize each column $D_{j}={\left[d_{1j},d_{2j},\text{…},d_{ij},\text{…},d_{mj}\right]}^{T}$ of the decision matrix D.

For the forward indicator (vaccination rate), the calculation for normalization can be expressed as:

(48) $$d_{ij}^{\prime }=\frac{d_{ij}-\min\left(D_{j}\right)}{\max\left(D_{j}\right)-\min\left(D_{j}\right)}.$$

For the reverse indicator (infection rate and case fatality rate), the calculation for normalization can be expressed as:

(49) $$d_{ij}^{\prime }=\frac{\max\left(D_{j}\right)-d_{ij}}{\max\left(D_{j}\right)-\min\left(D_{j}\right)}.$$

(3) Calculate the information entropy $e_{j}$ for each risk indicator j:
(50) $$e_{j}=-\frac{1}{\ln m}\sum_{i=1}^{m}p_{ij}\ln p_{ij},$$

where $p_{ij}=d_{ij}^{\prime }/\sum_{i=1}^{m}d_{ij}^{\prime }$.

(4) Determine the weight $w_{j}$ of each risk indicator j:
(51) $$w_{j}=\frac{1-e_{j}}{\sum_{j=1}^{3}\left(1-e_{j}\right)}.$$
(5) Calculate the comprehensive score $s_{i}$ of each airport i as its risk value:
(52) $$s_{i}=\sum_{j=1}^{3}w_{j}d_{ij}^{\prime }.$$

Remark 3 It should be noted that the term $\ln p_{ij}$ in Equation (50) is meaningless when $p_{ij}=0$. Thus, we let $p_{ij}\;\ln p_{ij}=0$ if $p_{ij}=0$.

Remark 4 The weight of each risk indicator is between 0 and 1, and the sum of all weights is equal to 1. Hence, the risk value of each airport is also between 0 and 1.

## 7 COMPUTATIONAL EXPERIMENTS

In this section, some computational experiments are conducted to utilize the mathematical model in actual cases and analyze the performance of the proposed algorithms. We first present a set of experiments to demonstrate the effectiveness of our proposed algorithms and perform the sensitivity analysis. Then, more complicated real‐world examples of India and the United States, representing the medium‐ and large‐scale experiments, respectively, are given to examine the solution efficiency and further discuss. From the methodological level, we compare the proposed Lagrangian relaxation approach with an MILP solver based on the branch‐and‐bound search (Henry et al., 2022). From the practical level, we illustrate the effectiveness of the air travel bubble strategy by comparing it with a benchmark strategy. All algorithms are implemented in MATLAB R2020b, and all numerical instances are tested on a personal computer with 1.8 GHz Intel(R) Core(TM) i7‐10510U CPU, 16.0 GB RAM, and Windows 10 64‐bit OS.

### 7.1 Parameter settings of the algorithm

The parameter settings of our Lagrangian relaxation‐based solution algorithms are presented as follows. The initial values of all Lagrange multipliers $\lambda_{i}$ are set to 0, and the iterative process starts with $UB=10^{7}$ and LB=0. The step‐size multiplier Δ is set to 2, and the number of consecutive iterations with no improvement in the lower bound LB is 30. This means if the lower bound LB fails to be improved in 30 consecutive iterations, the step‐size multiplier Δ will be halved. For terminal parameters, we set the optimality tolerance ε to 0.01 and the maximum number of iterations to 1000.

### 7.2 Performance analysis on a small network

**FIGURE 3:**
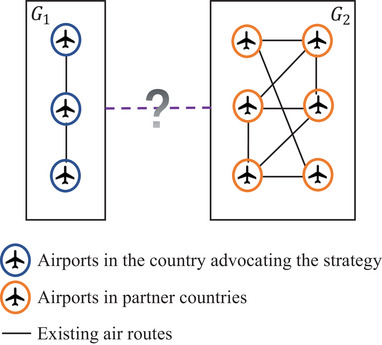
A hypothetical small case.

In this section, we consider a hypothetical small case shown in Figure 3. It can be seen that there are two small subnetworks $G_{1}$ and $G_{2}$, representing the airport connections in the country advocating the policy and its partner countries, respectively. We suppose each airport in $G_{1}$ and $G_{2}$ was directly connected at the prepandemic stage, which indicates that there are 36 OD pairs and 18 candidate links in this case. The prepandemic traffic demand of each OD pair is randomly generated from a number within [500,1000]. Each candidate link is set to a random capacity of 1000 or 1500. In addition, the risk value is also a random number from 0 to 1. Considering that transfer will cause much longer waiting times and produce a chain reaction if the previous flight is delayed, we set the maximum number of links on each path to 4. A reduction rate ρ is introduced to depict the decrease in traffic demand of each OD pair during the pandemic, which is equal to the reduction of traffic demand divided by the prepandemic demand. For characterizing the different degrees of demand decline and resource supply, we consider 18 combinations structured from setting the reduction rate ρ=80%,70%,60%, and the number of the restored links B=4,5,6,7,8,9. To better exemplify the experimental setup, we use an instance index, that is, *ρ*‐*B*, where these letters denote the reduction in demand and the available budgetary resources of each instance, respectively.

Table 4 presents the computational results of the instances with different values of ρ and B. For the experiments of the Lagrangian relaxation‐based approach, the column marked “UB” and “LB,” respectively, denote the final objective value of the primal and relaxed problem at the termination of the algorithm. The column labeled “Gap (%)” provides the smallest relative gap, and “Iter.” indicates the total number of Lagrangian iterations. For experiments of the MILP solver, the column marked “Objective” shows the value of the objective function, and the column labeled “Status” represents the current optimization status for the model. Two columns marked “Time (s)” display the total computational time in seconds for obtaining the solution in different approaches.

**TABLE 4:** Performance comparison of Lagrangian relaxation and MILP solver in the small case.

		Lagrangian relaxation					MILP solver		
ρ	B	UB	LB	Gap (%)	Iter.	Time (s)	Objective	Time (s)	Status
80%	4	4653.58	4612.68	0.8790	68	1.25	4653.58	0.63	OPTIMAL
	5	4069.46	4041.91	0.6770	163	1.32	4069.46	0.51	OPTIMAL
	6	3845.48	3808.52	0.9612	177	1.52	3845.48	0.59	OPTIMAL
	7	3654.74	3624.17	0.8363	122	1.20	3654.74	0.52	OPTIMAL
	8	3522.56	3501.36	0.6017	76	0.66	3522.56	0.46	OPTIMAL
	9	3411.16	3404.19	0.2045	130	1.44	3411.16	0.48	OPTIMAL
70%	4	–	–	–	–	–	–	–	INFEASIBLE
	5	–	–	–	–	–	–	–	INFEASIBLE
	6	6158.55	6097.10	0.9978	152	1.57	6158.55	0.61	OPTIMAL
	7	5720.41	5687.61	0.5732	61	0.58	5720.41	0.54	OPTIMAL
	8	5418.38	5399.00	0.3577	91	0.87	5418.38	0.52	OPTIMAL
	9	5217.28	5192.28	0.4793	172	1.73	5217.28	0.50	OPTIMAL
60%	4	–	–	–	–	–	–	–	INFEASIBLE
	5	–	–	–	–	–	–	–	INFEASIBLE
	6	–	–	–	–	–	–	–	INFEASIBLE
	7	–	–	–	–	–	–	–	INFEASIBLE
	8	7755.17	7681.23	0.9535	143	2.73	7755.17	0.56	OPTIMAL
	9	7308.97	7235.96	0.9988	164	1.34	7308.97	0.53	OPTIMAL

From Table 4, we have two major findings:

(1) For all 18 instances, the result of our Lagrangian relaxation‐based approach is the same as that using the MILP solver. Taking the optimization status in the solver as a benchmark, we can verify that our approach can accurately yield an optimal solution or find no feasible solution in this small case. In addition, it can be seen that regardless of the change in ρ and B, all instances can be solved in a relatively short time, which can demonstrate that the performance of our Lagrangian relaxation‐based algorithm is robust with the variation of the reduction rate and the budgetary resources.
(2) In these small‐scale experiments, the Lagrangian relaxation‐based algorithm takes a little longer solution time than the MILP solver, which is consistent with the results in Yun et al. (2015) and Zhao et al. (2018). The reason is that in each iteration of the Lagrangian relaxation‐based algorithm, we need to ensure that the current solution satisfies the total capacity constraint (see Algorithm 2). If not, a branch‐and‐bound approach will be embedded to solve the integer programming problem (34)‐(37). This will lead to a longer solution time. For example, the instances 80%−4 and 80%−5 have a similar solution time, but the number of iterations is different. In the instance 80%−4, 63 of the 68 iterations used the branch‐and‐bound technique to solve the problem (34)‐(37). On the contrary, the total capacity constraint holds for each iteration of the instance 80%−5, so we do not need to embed the branch‐and‐bound method during the solution process.

**FIGURE 4:**
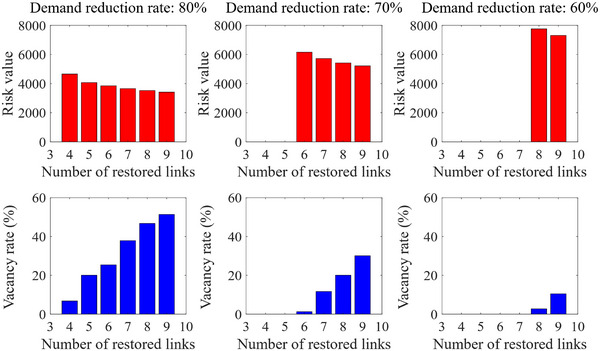
The variation of the risk value and the vacancy rate in small cases.

To examine how the key parameters affect the objective value of the ATBP, Figure 4 illustrates the variation of risk value in different parameter combinations. We also introduce a vacancy rate to reflect the utilization of the link capacity in Figure 4, equal to the actual carrying flow divided by the total capacity of the selected candidate links. We first analyze the impact of the available budgetary resources. As shown in Figure 4, the total risk value will decrease slightly if there are more restored links. Essentially, providing more air routes can increase end‐to‐end accessibility, and travelers can reduce the number of transfers for some OD pairs. This results in a lower risk of infection that passengers may encounter on the way. However, it is shown that the vacancy rate increases fast when the number of restored links rises. Indeed, the higher vacancy rate indicates a lower utilization of resources, but it may guarantee social distancing in the airplane, which is an essential preventive measure during pandemics (Salari et al., 2022). Therefore, it is crucial to comprehensively consider various solutions with different network design scenarios when determining the recovery scheme.

Observation 1 Enhancements in travel options through restoring more air routes lead to the diminishment of infection risk but result in more idle resources.

From Figure 4 and Observation 1, the decrease in infection risk is accompanied by an increase in vacancy rate with the same travel demand. Thus, it could be an interesting policy issue in the future to evaluate to which degree the pandemic requires empty seats in the airplane to increase the physical distance between passengers. Moreover, our finding echoes the guidance for air travel during the COVID‐19 pandemic issued by the International Civil Aviation Organization, which states that “seats should be assigned for adequate physical distancing between passengers when needed” and “airlines should allow for separated seating arrangements when occupancy allows it.”^6^

Meanwhile, the impact of the reduction in traffic demand is also evaluated. We can find that the minimum number of restored links will increase significantly with the decrease in the reduction rate. When ρ decreases from 80% to 60%, the upward trend of the minimum B value that provides a feasible solution to the ATBP is evident. It brings implications for policymakers that the schedule and planning of air routes in pandemics should be adapted to the changeable travel demand.

Observation 2 A higher demand level brings out larger investments in restored air routes.

Actually, the demand‐driven flight adjustments not only apply to the air traffic recovery during pandemics but are also used in regular aviation operations management. For instance, many airlines in the Middle East deploy extra flights to ensure more air connectivity for pilgrims during the Hajj season.^7^ Similar practices can be found in China for meeting the growing travel demand during the Spring Festival travel rush every year.^8^

### 7.3 Real‐world case study

#### 7.3.1 Data preparation

For our case study, we choose India as an example because India is one of the first to establish air travel bubbles with other countries after the COVID‐19 pandemic. According to the Ministry of Civil Aviation, India created air travel bubble arrangements with 13 countries as of September 2020, including “Afghanistan, Bahrain, Canada, France, Germany, Iraq, Japan, Maldives, Nigeria, Qatar, the UAE, the UK, and the USA.”^9^ The flight data set employed in this study is obtained from “Official Airline Guide (OAG).”^10^ We employ the origin airport, destination airport, seating capacity, and time series of each flight record. Therein, the flights between India and the above‐mentioned countries from October 1, 2019, to October 7, 2019, are selected as the prepandemic baseline. The flights in the same period of 2020 are used to design the air travel bubble strategy. Note that if the adopted flight data of 2020 contain the flights connecting India and its partner countries, we artificially exclude them in light of the problem description.

According to statistics, there are a total of 242 OD pairs between India and its partner countries at the prepandemic stage. The traffic flow of each OD pair is assumed to be the sum of the seating capacity of all corresponding direct flights. We suppose that the reduction rate ρ is 80%, which means the traffic demand $q_{i}$ of each OD pair during the pandemic is 20% of its traffic flow at the prepandemic stage. The number of international candidate links between $G_{1}$ and $G_{2}$ is 120, and the capacity $c_{l}$ of each candidate link during the pandemic is set as half of its prepandemic capacity for reserving empty seats. Considering the budgetary resources are very limited during the pandemic, the number of the restored international links B is set to 20. Each link carries a risk value equal to the higher risk of two corresponding airports, and the airport risk is determined by the EWM presented in Section 6. However, we do not have detailed information regarding the three risk indicators of all involved cities. Therefore, in this experiment, we investigate the distribution of confirmed cases in all U.S. states over 1 week and then use it to model the difference in infection risk.^11^ Based on this, a random number generator is used to generate the risk value of each airport.

#### 7.3.2 Results

**FIGURE 5:**
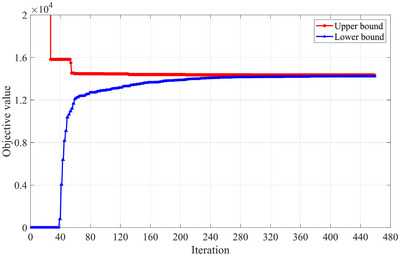
Best upper bounds and lower bounds in iterations (Case of India).

Based on the experimental setup, it is shown that the scale of this case is obviously larger than that in Section 7.2. To perform an efficiency comparison, we also use a time limit of 10 h for both the proposed Lagrangian relaxation approach and the MILP solver. The Lagrangian relaxation‐based algorithm iterates 459 times before triggering the termination condition of the relative gap, and the computation time is 14.84 s. However, for the MILP solver, it takes 5766.37 s to find the optimal solution. We can see that the advantage of the Lagrangian relaxation‐based algorithm becomes noticeable when the instance scale increases. It can solve the problem to a high‐quality solution within a much shorter solution time. The convergence process of the Lagrangian relaxation‐based algorithm is shown in Figure 5, which plots the best upper bounds and lower bounds in iterations. It can be found that these two bounds get closer rapidly before the 80th iteration, but the convergence tendency slows down after the 240th iteration. The upper bound is improved fast in the first 55 iterations, followed by a slower change in the subsequent iterations until termination. The lower bound remains at 0 before the 39th iteration due to the initial setting, implying the objective value of the relaxed problem in these iterations is less than or equal to 0. Then, it rises quickly from the 39th iteration to the 59th iteration and still grows at a slower pace after the 60th iteration. When the algorithm terminates in the 459th iteration, the relative gap is 0.79%.

**FIGURE 6:**
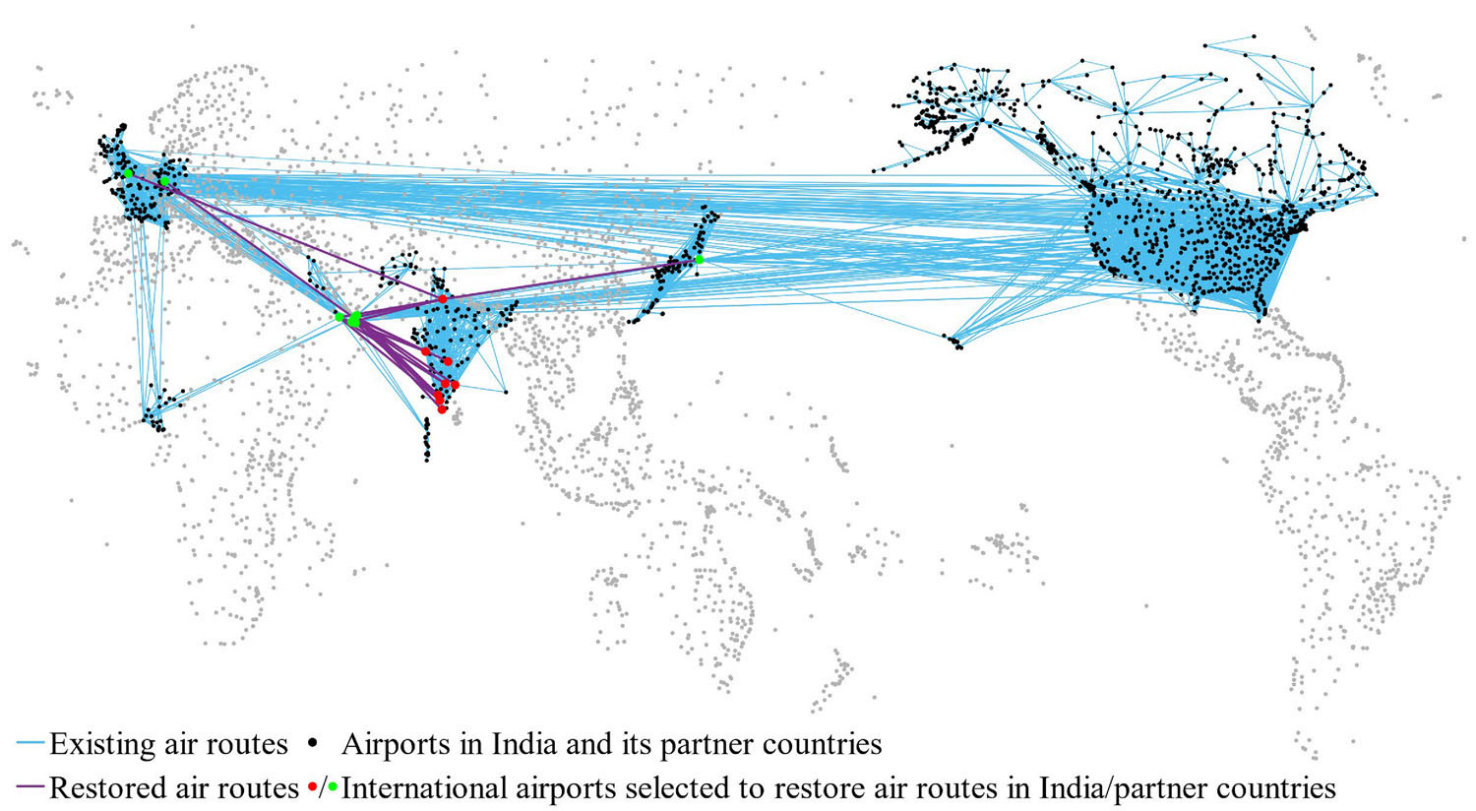
The geographical layout of the air travel bubble strategy (Case of India).

Figure 6 illustrates the existing and restored air routes under this air travel bubble strategy, represented by blue and purple links, respectively. In Figure 6, the red nodes denote the airports selected to restore international air routes in India, while the green nodes represent the corresponding airports in its partner countries. The detailed information on the restored air routes is listed in Table 5, where the left‐hand side of air routes represents the airports in India, and the right‐hand side denotes the airports in partner countries. From Figure 6 and Table 5, we see that most of these 20 restored air routes connect India with airports in the Middle East and Europe. There are two reasons to explain this phenomenon. On the one hand, the traffic flow of these air routes at the prepandemic stage is comparatively high, which leads to an adequate capacity reserve during the pandemic. On the other hand, airports chosen in its partner countries are mostly hub airports with a developed ATN. Thus, transfers are convenient to be made at these airports to enable air connectivity with other partner countries. Taking DXB (Dubai International Airport) as an example, it not only has high traffic demand with many airports in India but also directly connects with airports in some other countries which have not resumed direct air routes with India. It is interesting to find that the air routes CCJ‐AAN and CCJ‐RKT are decided to be restored even if their capacities are not very large. This is because CCJ (Calicut International Airport) is the only access in India to AAN (Al Ain International Airport) and RKT (Ras Al Khaimah International Airport), and other candidate air routes are unavailable to satisfy this traffic demand. Furthermore, the airports chosen for recovering air routes in India are widely distributed, which is helpful to relieve the pressure on large airports in epidemic prevention due to the high number of international flights.

**TABLE 5:** The information on the restored links (Case of India).

Index	Restored air route	Capacity	Traffic flow	Index	Restored air route	Capacity	Traffic flow
1	BOM‐DXB	27,109	27,109	11	COK‐DOH	5749	5491
2	DEL‐DXB	19,621	19,392	12	TRV‐SHJ	5616	3212
3	HYD‐DXB	12,098	12,098	13	CCJ‐SHJ	3938	3938
4	COK‐DXB	10,791	10,791	14	COK‐SHJ	3840	3840
5	DEL‐AUH	10,707	10,707	15	DEL‐NRT	3733	3733
6	MAA‐DXB	10,490	10,490	16	BOM‐SHJ	3654	3654
7	BOM‐AUH	9768	9768	17	BLR‐FRA	2548	1149
8	DEL‐LHR	8537	4904	18	BLR‐DOH	2478	992
9	DEL‐DOH	6334	4064	19	CCJ‐AAN	372	148
10	BOM‐DOH	5826	5826	20	CCJ‐RKT	372	148

Observation 3 Under the air travel bubble strategy, the restored air routes usually have greater capacity and are geographically dispersed.

The above analysis highlights the importance of decentralizing international air routes in different airports during pandemics. A counter‐example of the 2022 COVID‐19 outbreak in Shanghai can further illustrate the necessity. In Spring 2022, Shanghai experienced a nearly 2‐month city lockdown induced by the Omicron variant, seriously impacting the daily life of 25 million people. The main source of the virus for this outbreak was a quarantine site, where plenty of inbound travelers were quarantined.^12^ Recall that Shanghai is one of China's largest international air transport hubs, handling around 40% of China's international arrivals since the COVID‐19 pandemic began.^13^ Hence, the epidemic control pressure in Shanghai is relatively high due to the large number of imported cases. From this example, it can be found that concentrating too many international air routes in one place could cause widespread virus transmission, leading to substantial socioeconomic losses.

#### 7.3.3 Additional experiments

The same data set supposing the United States adopts the travel bubble strategy is also experimented to demonstrate our algorithms’ performance. There are a total of nine countries having direct international flights with the United States at the prepandemic stage, and the number of OD pairs is 735. We still assume that the traffic demand of each OD pair during the pandemic is 20% of its prepandemic traffic flow. The number of international candidate links is 368, and 70 of them will be chosen for restoration. We can find that this experiment has a much larger scale than all previous experiments.

**FIGURE 7:**
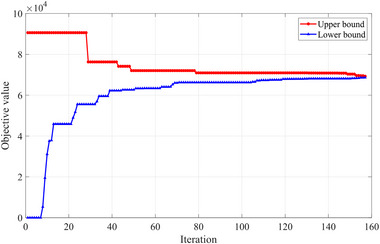
Best upper bounds and lower bounds in iterations (Case of the United States).

As shown in Figure 7, it takes 157 iterations to find the network design solution within a 0.98% relative gap using the Lagrangian relaxation‐based algorithm. The computation time in this process is 83.31 s, and the final objective value is 69329.03. Note that the upper bound changes less frequently than the lower bound, improving 12 times in all. It means that the upper bound is lowered for a total of 12 times during the solution process. Conversely, the lower bound shows a continuous upward trend, which is very similar to that in the case of India. To be specific, it grows rapidly at first, then slows down until it converges with the upper bound. Meanwhile, we also try to solve this problem with the MILP solver. However, we find that the MILP solver had difficulty in solving this instance because it is unable to provide a high‐quality solution in 10 h. The objective value obtained by using the MILP solver is 72347.59, which is much higher than that gained from the proposed Lagrangian relaxation approach. Therefore, we believe that our proposed Lagrangian relaxation‐based algorithm performs better than the MILP solver for the ATBP, especially in large‐scale cases.

**FIGURE 8:**
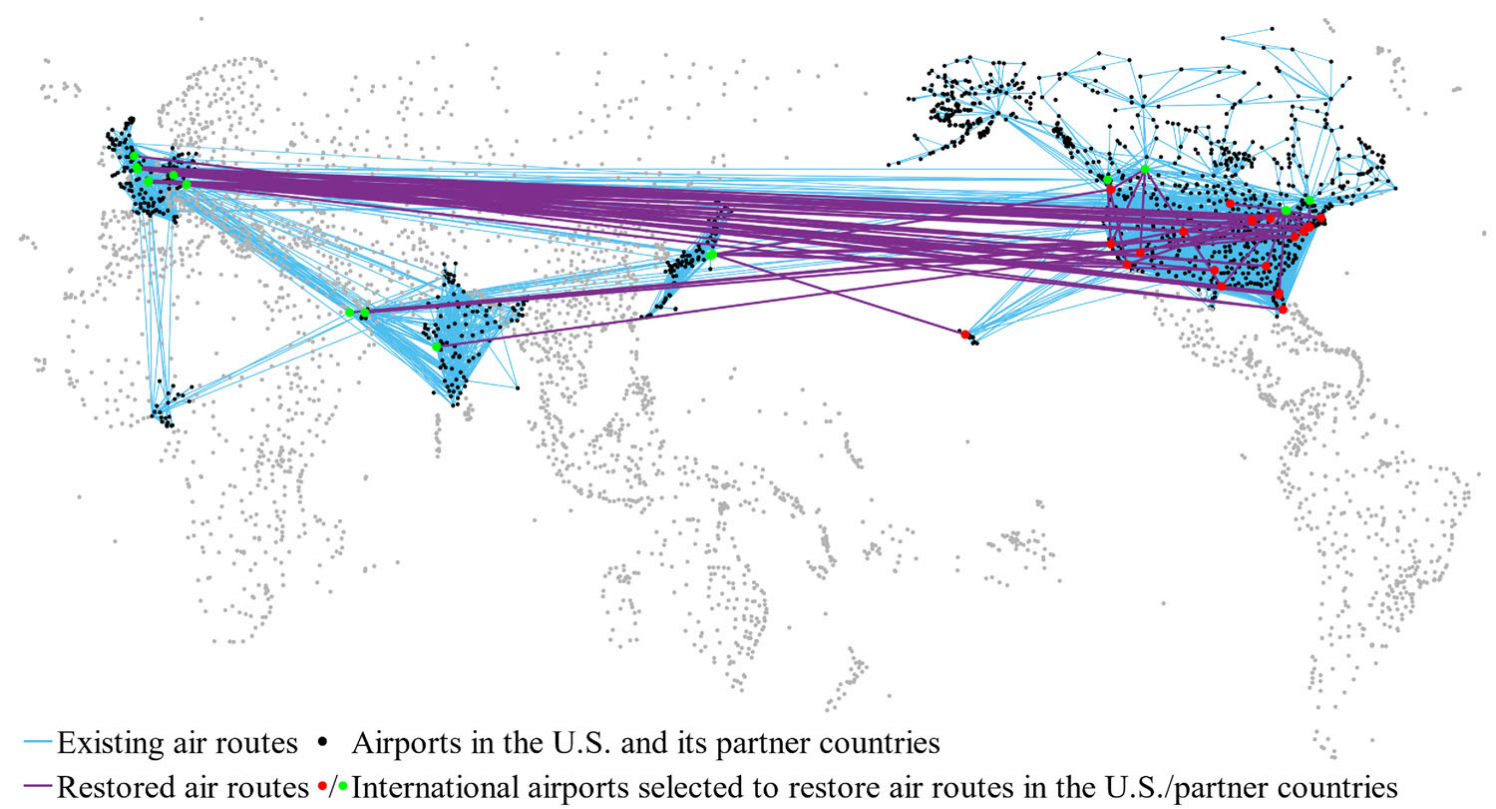
The geographical layout of the air travel bubble strategy (Case of the United States).

Similarly, we present the geographical layout of the air travel bubble strategy in Figure 8 based on our solution results. It can be seen that the restored air routes of the United States cover most of its partner countries, which is quite different from the layout of India. One apparent reason is that the United States has a wide distribution of international traffic demand compared to India. In addition, from the case of the United States, we can find that the restored air routes connecting Canada and Europe are an order of magnitude greater than those linking other countries. For instance, among all 22 international airports selected to restore air routes in the United States, 15 airports are resuming air routes with YYZ (Toronto Pearson International Airport) and nine airports with CDG (Paris Charles de Gaulle International Airport). It reveals that airports with high and wide traffic demand in partner countries before the pandemic are still immensely favored in the decision of the travel bubble strategy.

To sum up, although the same data set is used for the Indian and U.S. cases, the recovery schemes obtained in the ATBP are divergent. The traffic demand, budgetary resources, and partner countries of India and the United States are quite different, which would directly influence the restored air routes and the in‐transit infection risk. Moreover, the recovery schemes are also relevant to the aviation restoration orientation of different countries. India primarily focuses on restoring trunk lines with its partner countries, while the United States adopts a more widespread approach. Overall, these differences underscore the need for tailored and context‐specific strategies in the implementation of travel bubble policies by each country.

#### 7.3.4 Effectiveness

To characterize the effectiveness of the proposed air travel bubble strategy, we present a traditional air transport recovery strategy as a benchmark for further comparisons. This benchmark strategy determines the restored international air routes based on the travel demand from each partner country. First, the candidate air routes between the leading country and each partner country are sorted in descending order according to their travel demand, and the first B routes will be restored. Then, a distance‐based model is applied to assign the traffic flow. The detailed route sorting rule and traffic assignment model are provided in Appendix B.

**FIGURE 9:**
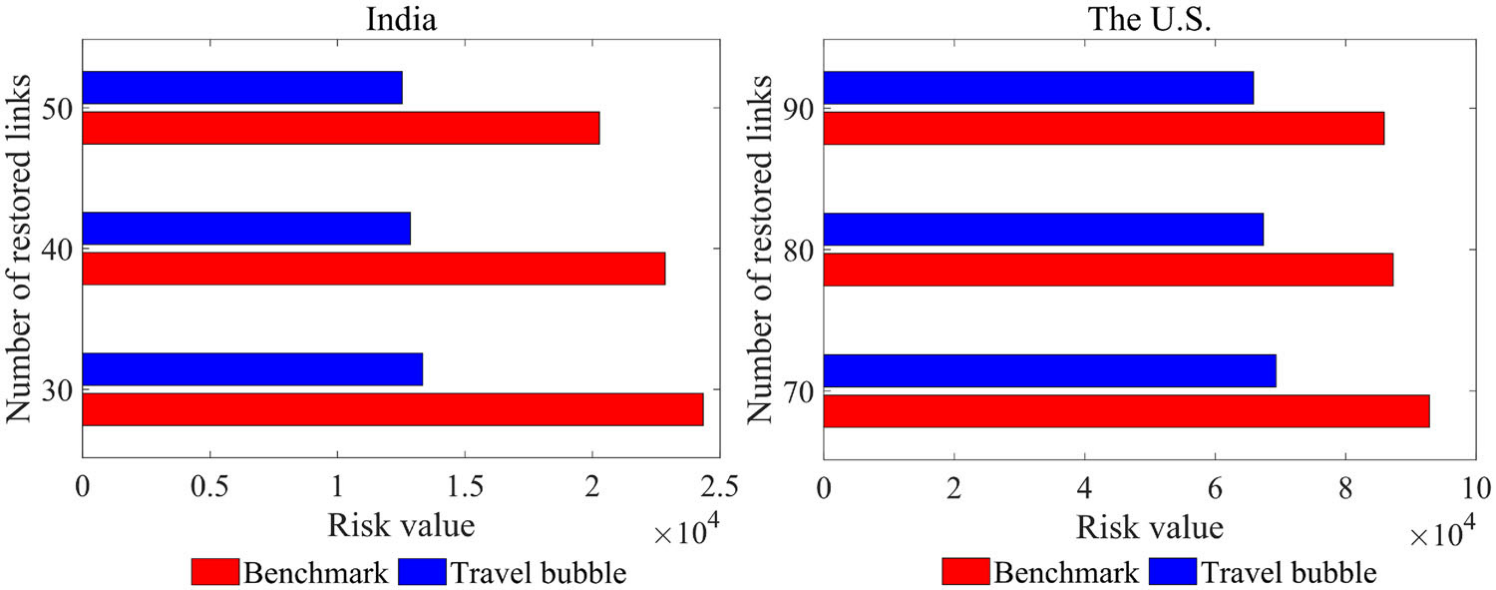
The total infection risk under the benchmark strategy and the air travel bubble strategy.

We compare the total infection risk under the benchmark and air travel bubble strategies in the real‐world cases of India and the United States. The risk values with different numbers of the restored international links B are shown in Figure 9. It can be seen that our air travel bubble strategy exhibits evident effectiveness in terms of preventing infections in all scenarios. Compared to the benchmark strategy, the maximum reduction rate in infection risk is 45.2%. We can also find that the risk reduction in India is higher than that in the United States. A possible conjecture is that the greater demand for international travel leads to a higher value of total infection risk in the United States. Therefore, no matter how the ATN is designed, the infection risk will be maintained at a certain level.

Observation 4 With the same recovery resources, the air travel bubble strategy substantially outperforms the benchmark strategy since it can reduce the total infection risk by 22.8%−45.2% in our tested instances.

To summarize, we see that the air travel bubble strategy can effectively mitigate the infection risk exposed to passengers and, therefore, expectedly enhance the passengers’ travel experience. In particular, when the number of restored air routes is at a low level, closer to the condition of fewer budgetary resources during pandemics, the extent of risk reduction is more pronounced.

## 8 DISCUSSIONS

In this section, we discuss the theoretical and practical implications.

### 8.1 Theoretical implications

Our study extends the current literature on travel bubble policies from the perspective of risk‐oriented strategy design. Previous studies indicate that the travel bubble policy is a viable solution for reviving international air travel during pandemics (Chen et al., 2023; Fusté‐Forné & Michael, 2023; Sun et al., 2022), but the effectiveness and safety hinge heavily on meticulous planning and coordination among governments. Therefore, a formal mathematical framework is proposed to model the ATBP with the aim of minimizing the in‐transit infection risk. In addition, we also present a risk evaluation approach based on the EWM, which has the flexibility to take into account different pandemic‐related decision‐making indices. The case study of India and the United States shows that the proposed travel bubble strategy can control the infection risk during the air traffic restoration process, and it outperforms a demand‐driven recovery strategy in all scenarios with different budgetary resources and travel demands.

Another theoretical implication is on the transportation network design, considering subnetwork connections. During pandemics like COVID‐19, the phenomenon of country‐to‐country disconnections due to several entry restriction policies becomes a pervasive feature in ATNs (Li et al., 2021; Wang et al., 2022). Hence, we construct a novel link‐based network design model to characterize the connections between subnetworks, which can fulfill the travel demands of all OD pairs while minimizing the total infection risk on all links. To efficiently solve the problem, this link‐based model is further transformed into a path‐based model. Then, the Lagrangian relaxation‐based solution algorithm is proposed to quickly obtain high‐quality solutions. To sum up, our study offers a systematic framework that empowers policymakers with a comprehensive decision‐making tool for air traffic restoration. It also has the potential to enhance the resilience of ATNs in navigating the complex environment during pandemics.

### 8.2 Practical implications

Based on the scientific results, our study also provides some practical implications on the pandemic‐related air transport management. We discuss them as follows.

The first practical implication is that prioritizing public health and safety by considering infection risk is paramount when determining air transport recovery schemes. Our analysis shows that the proposed risk‐oriented travel bubble strategy can significantly reduce the infection risk while fulfilling the travel demand. In addition, the risk value of different schemes can provide useful guidance to the selection of budgetary resources, which is an important prerequisite for the construction of travel bubbles. All of these findings imply that the air travel bubble policy should be implemented based on realistic risk considerations. Specifically, policymakers and aviation operations regulators should carefully examine whether it is necessary to construct a safe bubble according to the current pandemic situation and whether the balance between mitigating the infection risk and satisfying the travel demand can be made.

The second implication highlights the importance of critical airports in partner countries, which play a fundamental role in the air travel bubble policy. The findings in our case study illustrate that the air travel recovery strategy tends to restore air routes with those airports that not only have a large quantity of bilateral traffic demand but also are global hubs with high international connectivity. Examples include DXB (Dubai International Airport) in the case of India and CDG (Paris Charles de Gaulle International Airport) in the case of the United States. Therefore, the leading country should pay more attention to these critical airports in their partner countries. Partnerships must be carefully selected and well maintained to reserve the binding link capacity during pandemics.

The third implication relates to the layout of ATNs, which is a necessary guarantee for implementing the air travel bubble policy. The results of our work suggest that both for India and the United States, the layout of ATNs makes the location of the entry and exit for passengers dispersed in different areas of the leading country. In detail, the airports selected for resuming the international air routes are scattered, and the restored routes are not just concentrated on certain airports. The above findings highlight the implication that it is significant to divert international air routes to different airports across the country during pandemics. This measure can benefit from distributing the pressure of pandemic prevention and reducing virus transmission.

## 9 CONCLUSIONS

Air transport recovery is indispensable in low‐incidence regions during pandemics, as it can supply the essential travel demand and improve the connectivity of ATNs. This article investigates a novel policy called the “travel bubble” for restarting international aviation during pandemics. Mathematical models are constructed to describe the risk‐oriented ATBP, and effective solution algorithms are developed. The optimization framework provides a reference for aviation operations regulators when considering establishing travel bubbles with other countries. To the best of our knowledge, this study is the first to determine the air route restoration and passenger flow assignment under the travel bubble policy during pandemics.

To reflect the key features of air transport recovery during pandemics, a link‐based network design model is first built for the ATBP, which considers the link connection of disconnected networks with the minimal infection risk for satisfying all specific travel demands between the leading country and its partner countries. Then, it is further reformulated as a path‐based model based on the property of this problem, which reduces plenty of variables and constraints for ease of solving. Finally, a Lagrangian relaxation‐based solution framework is proposed to yield the optimal solutions. Experimental results illustrate that the developed solution algorithm effectively solves the network design model. Moreover, it can obtain high‐quality solutions with a small optimality gap in a short computational time. It also outperforms an MILP solver for medium‐ and large‐scale instances, which shows that it is an efficient tool for real‐world applications. Apart from providing a powerful decision support tool to determine recovery schemes for low‐risk air transport recovery, some practical insights and implications for policymakers and aviation operations regulators are also drawn from our computational experiments. We hope that these insights will be effective on guiding air transport management in emergency situations.

Although the travel bubble policy has been conducted during the recent COVID‐19 pandemic, our optimization method can be applied to aviation recovery decisions for future public health emergencies and pandemics of different types. Moreover, with the objective of minimizing infection risk, our method can be extended to help policymakers evaluate which countries are suitable for travel bubble cooperation during pandemics. A broader idea is to introduce a set of potential partner countries based on our problem. Thus, a new category of decision variables can be added to indicate whether a country is selected for constructing the air travel bubble. Last but not least, it is worth mentioning that the “bubble” idea can also be utilized in other scenarios beyond pandemics for risk control. For instance, during natural disasters like earthquakes and hurricanes, establishing point‐to‐point transportation bubbles can facilitate the safe evacuation of affected people. The designated evacuation routes between disaster areas and temporary shelters can reduce the in‐transit risk to evacuees and improve the efficiency of evacuation.

Future extensions for this study may be possible in the following three aspects. First, the inherent uncertainties in some parameters of our model, such as the airport risk and the travel demand during pandemics, could be considered. Second, some other factors, like the total travel cost, could be included in the objective, and a multi‐objective optimization or artificial intelligence (Nguyen et al., 2023) approach could be used to construct a more complex travel bubble model. Third, the optimal recovery sequence of the selected international air routes in the travel bubble could be investigated in the future.

## Footnotes

1 What is a Travel Bubble. https://blog.wego.com/whats‐a‐travel‐bubble/ (accessed March 26, 2023).

2 Trans‐Tasman bubble lifts travel between New Zealand, Australia. http://www.xinhuanet.com/english/asiapacific/2021‐06/14/c_1310007094.htm (accessed November 8, 2023).

3 Tourism New Zealand forecasting billion‐dollar economy boost if trans‐Tasman bubble opens. https://www.rnz.co.nz/news/national/439782/tourism‐new‐zealand‐forecasting‐billion‐dollar‐economy‐boost‐if‐trans‐tasman‐bubble‐opens (accessed November 8, 2023).

4 Restarting international aviation through “travel bubbles.” https://www.iata.org/contentassets/5c8786230ff34e2da406c72a52030e95/restarting‐international‐aviation‐through‐travel‐bubbles.pdf (accessed March 26, 2023).

5 Additionally, there are some other indicators that also affects the infection risk, such as the basic reproduction number of the disease, the efficacy rate of vaccines, and the number of fake vaccination cards. Due to data limitation concerning the above indicators, we have not considered them in the current work. Hence, future research may aim at considering those additional indicators which may improve the accuracy of the risk evaluation.

6 Guidance for air travel through the COVID‐19 public health crisis. https://www.icao.int/covid/cart/Pages/CART‐Take‐off.aspx (accessed June 23, 2023).

7 Emirates gears up for busy Hajj season with extra flights to Jeddah & Medina. https://simpleflying.com/emirates‐extra‐hajj‐flights‐jeddah‐medina/ (accessed June 23, 2023).

8 30,000 extra flights OK'd for Spring Festival. https://global.chinadaily.com.cn/a/201801/31/WS5a71c933a3106e7dcc13a061.html (accessed June 23, 2023).

9 Ministry of Civil Aviation, https://www.civilaviation.gov.in/en.

10 Official Airline Guide (OAG), https://analytics.oag.com/.

11 Data source of confirmed cases: https://www.worldometers.info/coronavirus/country/us/.

12 Quarantine site blamed for COVID‐19 outbreak in Shanghai. https://www.shine.cn/news/metro/2203112995/ (accessed June 23, 2023).

13 COVID‐19 surge prompts China to divert flights away from Shanghai. https://simpleflying.com/covid‐19‐surge‐china‐divert‐flights‐from‐shanghai/ (accessed June 23, 2023).

## APPENDIX A PROOF OF **PROPOSITION 3**

We prove Proposition 3 by showing that the single allocation problem with fixed hubs, a known NP‐hard problem proposed in the literature such as Sohn and Park (2000), can be reduced to the air travel bubble problem (ATBP) in polynomial time.

The single allocation problem with fixed hubs can be stated as follows. Consider a single allocation hub network G with a set of nodes V. The hub nodes are fixed in the set (H⊆V), and the nonhub nodes are in the set $V_{0}=V-H$. All hubs are fully interconnected, and each nonhub node has to be connected to precisely one of the hubs. The amounts of flows and the unit costs of direct flows are given. Hence, the objective is to find an optimal allocation of nonhub nodes to the hubs, minimizing the total transportation cost. Sohn and Park (2000) proved that this single allocation problem with fixed hubs is NP‐hard when the number of the fixed hubs is larger than or equal to three.

We now establish a specification of the ATBP and depict it as a single allocation problem with fixed hubs. In the ATBP, we still consider two subnetworks $G_{1}$ and $G_{2}$, which have the same meaning as in Section 3. All airport nodes in $G_{1}$ is isolated, which means $|E_{1}|=0$. Conversely, all airport nodes in $G_{2}$ are fully interconnected, which means $G_{2}$ is a complete graph and $|E_{2}\left|=\right|V_{2}|\cdot \left(|V_{2}|-1\right)/2$. Note that we suppose $|V_{2}|\geq 3$. Thus, we can see that $V_{1}$ can be regarded as the set of nonhub nodes, while $V_{2}$ can be regarded as the set of hubs in the above‐mentioned single allocation problem. A total of $\left|V|=\right|V_{1}\left|+\right|V_{2}|$ airport nodes constitute the single allocation hub network G. We assume that all airport nodes in $V_{1}$ have direct connections with those in $V_{2}$ at the prepandemic stage, so the number of candidate links $|E_{3}\left|=\right|V_{1}\left|\cdot \right|V_{2}|$. Let the number of restored links B be $|V_{1}|$, and the capacity of each candidate link be infinite. In addition, the maximum number of links on each path T is set as 3, and the risk of each link is taken as the unit transportation cost. Here, it can be found that an optimal solution to the single allocation problem with fixed hubs is also an optimal solution for this special case of the ATBP described above. Therefore, the single allocation problem with fixed hubs is reducible to the ATBP.□

## APPENDIX B THE BENCHMARK STRATEGY

The benchmark strategy for air transport recovery is divided into two steps. The first step is the selection of restored air routes, and the second step is the assignment of traffic flow. For the first step, the respective strategy chooses air routes with higher travel demand and ensures that each partner country has direct flights with the leading country. The detailed procedure is presented in Algorithm B1. It continuously picks out the air route with the highest demand for each partner country, and finally chooses B air routes for recovery.

ALGORITHM B1 Algorithm for selecting the restored links in the benchmark strategy. **Table:**

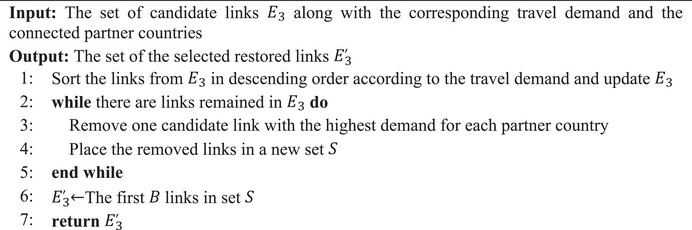

With the selected air routes, the optimal traffic assignment is determined based on the following mathematical model:

(B1)
$$\min\;\sum_{i\in I}\sum_{l\in E_{3}^{\prime }}q_{i}d_{il}z_{il},$$

(B2)
$$s.t.\;\sum_{l\in E_{3}^{\prime }}z_{il}=1\;\forall i\in I.$$

(B3)
$$\sum_{i\in I}q_{i}z_{il}\leq c_{l}\;\forall l\in E_{3}^{\prime }.$$

(B4)
$$0\leq z_{il}\leq 1\;\forall i\in I,l\in E_{3}^{\prime }.$$

In this model, $E_{3}^{\prime }$ denotes the selected international links, and $d_{il}$ represents the spherical distance of OD pair i∈I using the international link $l\in E_{3}^{\prime }$. The objective function (B1) minimizes the total spherical distance. Constraints (B2) require that the traffic demand of each OD pair should be served by its possible paths. Constraints (B3) state that the carrying flow of each international link cannot exceed its capacity. Constraints (B4) indicate the range of decision variables. After solving this model, the total infection risk R of the benchmark strategy can be calculated by:

(B5)
$$R=\sum_{i\in I}\sum_{l\in E_{3}^{\prime }}q_{i}r_{il}z_{il},$$

where $r_{il}$ is the same unit risk in the air travel bubble strategy.
